# Beyond tsunami fragility functions: experimental assessment for building damage estimation

**DOI:** 10.1038/s41598-023-41047-y

**Published:** 2023-08-31

**Authors:** Ruben Vescovo, Bruno Adriano, Erick Mas, Shunichi Koshimura

**Affiliations:** 1 Department of Civil and Environmental Engineering, Tohoku University, Aoba 468-1, Aramaki, Aoba-ku, Sendai, 980-8572 Japan; 2 International Research Institute of Disaster Science (IRIDeS), Tohoku University, Aoba 468-1, Aramaki, Aoba-ku, Sendai, 980-8572 Japan

**Keywords:** Natural hazards, Civil engineering, Environmental impact

## Abstract

Tsunami fragility functions (TFF) are statistical models that relate a tsunami intensity measure to a given building damage state, expressed as cumulative probability. Advances in computational and data retrieval speeds, coupled with novel deep learning applications to disaster science, have shifted research focus away from statistical estimators. TFFs offer a “disaster signature” with comparative value, though these models are seldom applied to generate damage estimates. With applicability in mind, we challenge this notion and investigate a portion of TFF literature, selecting three TFFs and two application methodologies to generate a building damage estimation baseline. Further, we propose a simple machine learning method, trained on physical parameters inspired by, but expanded beyond, TFF intensity measures. We test these three methods on the 2011 Ishinomaki dataset after the Great East Japan Earthquake and Tsunami in both binary and multi-class cases. We explore: (1) the quality of building damage estimation using TFF application methods; (2) whether TFF can generalize to out-of-domain building damage datasets; (3) a novel machine learning approach to perform the same task. Our findings suggest that: both TFF methods and our model have the potential to achieve good binary results; TFF methods struggle with multiple classes and out-of-domain tasks, while our proposed method appears to generalize better.

## Introduction

Statistical methods, and machine learning informed by remotely sensed information, have taken center stage in recent works attempting to understand disaster-borne damage, its detection, and its estimation. Tsunami fragility functions are one such method, used in disaster research^1^, to model building damage after a tsunami. Essentially, these regression models map a tsunami intensity measure (in the form of a demand parameter, such as inundation depth) to the probability of exceeding a discrete damage state. The intensity measure is often parameterized by an observable measure of the disaster. The parameter of choice has quintessentially been maximum inundation depth, as it is immediately measurable after the disaster. Derived quantities, usually obtained via hydrodynamic modelling, can be alternatively used and have been subject to study^1–3^.

Though visually significant, it remains unclear how fragility functions can be applied pragmatically: can they be applied to new data in a predictive manner? Hence, can future damage inferences at the building scale be made using exiting fragility functions?

More recently, efforts in the field of building damage estimation have moved away from modelling damage as a function of a disaster intensity measure. Recent research favors innovations in the field of computer vision to perform change detection between pre and post event imagery such as^4,5^. Crucially however, these novel methods forgo damage estimation, and instead leverage faster availability of post event satellite imagery to perform damage detection. This departure from a physical description of the damage, precludes the model from ever learning from context.

In this article, we explore the application of tsunami fragility functions as damage estimators. In the framework of our experiments, we perform estimations for individual buildings as described in the literature. Noting TFF limitations and lessons learned from consulting the literature, we propose an additional framework, using machine learning, to perform the same task. We train our model based on intensity measures inspired by TFF studies, but with expanded dimensionality. We aim to contribute in the following capacity: (1) explore the differences between TFF application methods; (2) verify in what capacity, previously untested, TFF applications are transferable building damage estimators; finally (3) we propose a novel framework to perform building damage estimation using machine learning classifiers.

Our results suggest TFF application methods to building damage estimation struggle to generalize. However, our experiments with machine learning models show promising resilience and outperform TFF methods in multi-class out-of-domain scenarios. The structure of this paper is as follows: in the “Motive” section we explore the relevance and background of post disaster building damage estimation, hence identify research gaps. The “Results” section reports experimental outcomes following our proposed methodology and the benchmarks from the literature. We discuss our results which show that machine learning methods appear to generalize better to different domains and multiple classes.

We conclude by, once again, going over our main findings. Details regarding the dataset, feature extraction, and methodology are reported in “Methods” section.

## Motive

Disasters undoubtedly present some of the greatest modern challenges to humanity. As the urbanized world grows, so do the effects of disasters on society, whether directly (from loss of lives) or by cascading effects (such as loss of land). More recently, profound socio-economic impacts of disasters have driven research into all aspects of preventive and management measures. Research output centered on tsunami disaster risk management has experienced incredible innovation in the last two decades. Herein, we explore the background of some of the most popular methods to quantify and assess post-event tsunami damage.

### Tsunami fragility functions

Fragility functions are regression models that attempt to represent the relationship between a disaster intensity measure (IM) or engineering demand parameter (*EDP*) (the independent variable, modelled as a continuous random variable), and the structural response of buildings under the intensity load, i.e., the damage state, *DS* (the dependent variable, modelled as a probability of exceedance). While most scientists agree on this initial formulation, the choice of IM/EDP, model, and approach have been the subject of academic debate^3^. In the following sections, we explore some background focusing on TFFs as a whole, differences in formulation, applications, and limitations.

#### Background

Fragility functions have been widespread in earthquake engineering^6,7^, as a convenient means of characterizing local earthquake impact, and were adapted to tsunami damage^1,2^. Unlike seismic activity, measuring tsunami intensity directly is problematic. The only practically measurable quantity is the maximum inundation level, *z*, which is measured from inundation traces left on affected structures. Notably, this does not necessarily represent the water level at failure^8^. Moreover, the search for an optimal combination of demand parameters has been the subject several studies^3,9,10^.

At times even *z*, the single most featured EDP, may not be measurable at sufficient resolution (i.e., when experts are not available or RS data cannot be validated) and must be interpolated^8,10^ or otherwise estimated^1, 11,12^. The measured inundation depth is often used to validate numerical models that have adjustable resolution—these come with the added benefit of generating secondary hydrodynamic quantities that can be used as EDP (i.e., hydrodynamic force, velocity, momentum, unit-less factors, etc.)^1,3,11–20^ independently or in pairs, in order to generate “fragility surfaces”^16,17^. Building on these findings, Macabaug et al.^20^ investigate the quality of several EDP by ranking them based on their predictive error, using generalized additive models (GAMs). To address discrete and sparse data (such as building material and building age, etc.) authors have proposed several adaptations: for instance, by splitting the damage data and aggregating it in terms of structural characteristics, authors have attempted to reduce latent variability^9,10,17,18,20–25^. Topographic features^18,19,23,26^, and physical effects^10,17,19,20^ (i.e., geomorphology, debris, building arrangements and shielding, etc.) have been similarly parameterized, especially in more recent publications featuring generalized linear models (GLMs). Indeed, the choice of statistical model, method of fit, and statistical correctness have been the object of debate: ordinary least squares (OLS) is the most popular formulation^1,2,8,11–15,18,19,21,22,25–30^; it requires the data to be aggregated in some form. A linear function is then regressed to the aggregate mean of the sample. In most cases, this is the the proportion of buildings exceeding a discrete damage state in terms of a continuous EDP. GLMs (and GAMs) have been adopted in several instances^3,9,10,16,17,20,23,24,31^ and allow the direct fit of a linear model to discrete, disaggregated data. Of great concern to the present study, several post disaster domains have been studies and modelled using fragility functions. It is these studies that initially warn against the general application of TFF models suggesting domain dependence^8,21,27^. The literature covers both historical and contemporary events, from the inception of TFFs between 2005^2^ and 2009^1^, some prominent examples are: the 1993 Okushiri Earthquake^11^, the 2004 Indian Ocean earthquake and tsunami^1,2,12,31^, the 2009 Samoa earthquake and tsunami^10,13^, the 2010 Chile earthquake^8^, the 2011 Great East Japan earthquake (GEJE)^3,9,14–24,26,27,29,32,33^ which features extremely detailed survey and supporting data^34–36^, and the 2018 Sulawesi earthquake and tsunami^30,31,37^. Due to the lack of classifiable damage data, analytic studies usually define building damage as a function of generalized structural properties (i.e., stress and strain)^25,38^, design standards, and precedent (such as a the impact of a previous disaster). TFFs have not been limited to buildings: indeed, vegetation^15^, road^33^, vessel^16^, and service pole^37^ damage has been framed in the same fashion. The TFF research corpus has been reviewed several times: we point to Tarbotton et al.^39^ and Charvet et al.^40^ for the latest comprehensive reviews (up to the date of publishing). Behrens et al.^41^ provide a recent review and breakdown of research gaps in probabilistic tsunami hazard and risk studies, including tsunami fragility functions.

### Machine learning

Examples of machine learning applications to disaster damage detection, in particular tsunami, leveraged classification algorithms and remote sensing change detection techniques. Generally they associate (supervised) or discover (unsupervised) labels to specific thresholds of change usually given an input of image patches containing a single building each. Before the advent of deep learning, a variety of algorithms were proposed with varying efficacy, support vector machines^42^ and ensembles methods^43^ count among the more popular. Recently, advances in computer hardware allowed for deep artificial neural networks (ANN) to be trained in useful time enabling automatic feature extraction from remote sensing imagery^42^. Remote sensing change detection currently comprises the state of the art when it comes to computer vision applications for natural disaster science. However, to our knowledge none of these methods employ intensity measures associated with tsunami dynamics, such as those used in fragility function studies. We postulate that, reliance on photogrammetry alone does not allow a model to learn anything from the physical processes that cause the damage. These models are naive in the sense that they learn what damage looks like, rather than why damage looks the way it does. As such they fall outside of the scope of the present study.

### Research gaps

While reviewing gaps in physical vulnerability tsunami models (including fragility functions), Behrens et al.^41^ indicate *“[...] a lack of consensus on many aspects of physical fragility and vulnerability modeling”*. Above we briefly explored the research corpus concerned with fragility functions. Herein, we systematically report research gaps concerning the application of tsunami fragility functions as building damage estimators. There are relatively few studies that attempt to apply tsunami fragility functions: Musa et al.^44^ build a real time tsunami computation routine that incorporates tsunami building damage on a zonal level using pre-existing TFFs; Rehman and Cho^28^ explore a case study using a similar method, albeit not in real time, wherein they apply TFFs generated from the 2011 GEJE data to Imwon Port, South Korea by simulating various tsunami scenarios. Adriano et al.^45^ and Moya et al.^46^ apply fragility functions to real disaster data to estimate damage. The first study proposes hypothetical scenarios hence results are not evaluated against a ground truth, while the second study only reports the building damage ratio for each class against the true ratio. Ultimately the evidence suggests that; Tsunami fragility functions do not generalize: high resolution tsunami inundation is usually obtained by validating hydrodynamic simulations against measured values^1,20^; the modelling requires a high resolution input (including elevation, spatial distribution, roughness, building arrangement, etc.) which is ultimately synthesized and aggregated into measures of intensity. Reversing a fragility function does not resolve into the original data used high resolution data nor does it preserve the spatial distribution of the disaggregated data. While studies^1^ have assumed that an estimate of building damage numbers can be obtained from a fragility function, they also warn against the generality of these functions^1,8^. Considering the points raised above it is unlikely tsunami fragility functions can generalize to different domains for the purposes of building damage estimation. Survey data is relatively scarce: while there is an abundance of fragility functions, they often model the same events. Empirical studies are limited to events following the 2004 Indian Ocean Tsunami^41^; post event surveys are risky, expensive, and require specialized personnel; many countries that experience tsunami disasters may not have the resources or personnel to conduct high quality damage surveys. A model that estimates building damage must be able to learn damage representations from a relatively small amount of data. Current machine learning methods are not damage estimators: Fragility functions establish a cause-effect relation between an intensity measure (of a disaster) and a building damage state; This implication is necessary to make estimations about future scenarios; current deep learning methods based on remote sensing imagery do not incorporate any measure of the disaster, hence cannot make estimates of future events.

### Objectives

**Figure 1:**
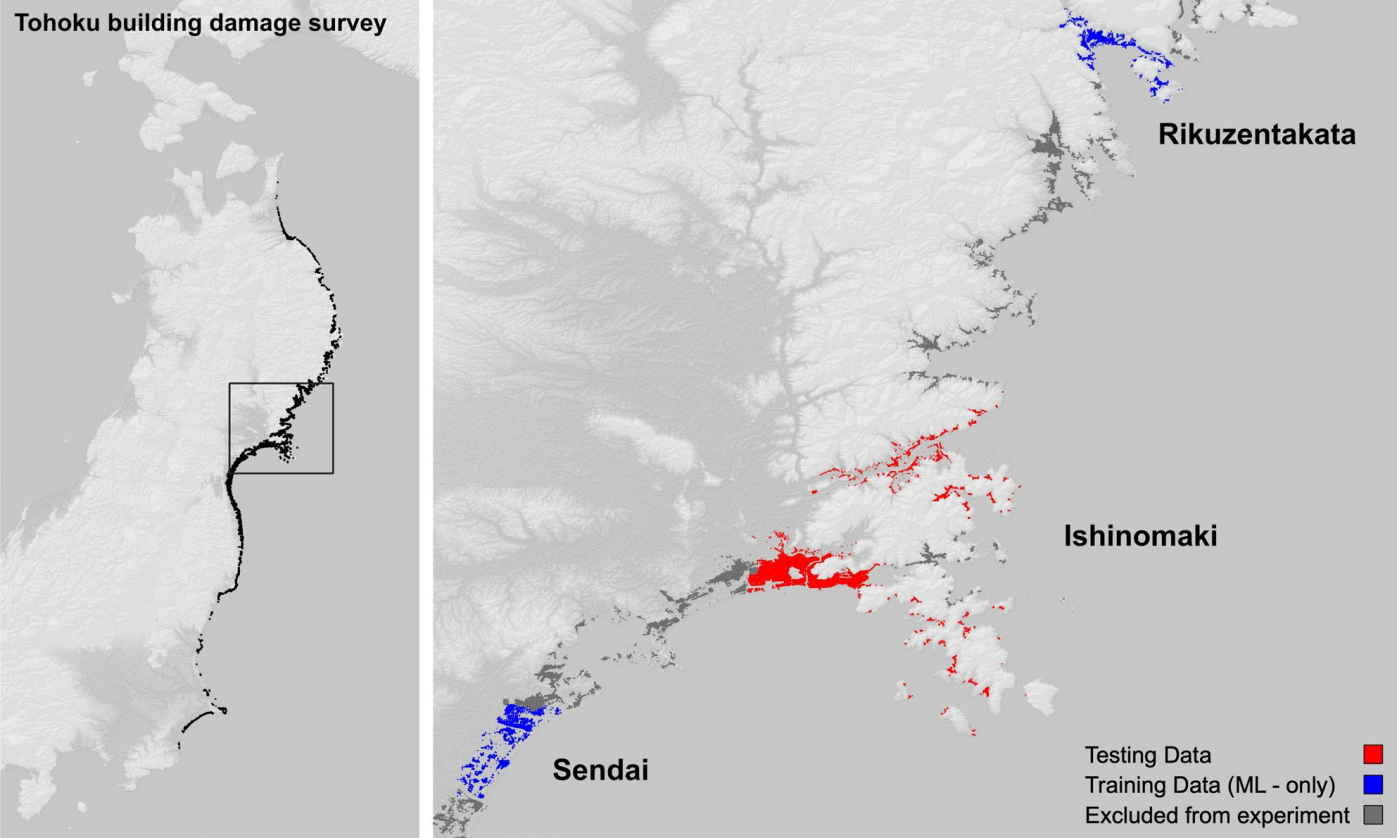
Left: building damage inventory following the 2011 Great East Japan Earthquake and subsequent tsunami^34,35^. Right: subsets of the dataset used in the present experiments; in blue: training data for the proposed ML approach; in red: testing data used for all experiments. Original maps generated using QGIS 3.28.2-Firenze (https://qgis.org/en/site); backdrop generated in QGIS from the Public Domain SRTM digital elevation model (10.5066/F7PR7TFT).

Many of the works^8,21,27^ that include a comparative analysis, point to the differences between the newly built function and previous functions when characterizing the unique tsunami damage. That is, the damage as a function of the set of demand parameters is specific and is explained by the demand parameters intrinsically. Intuitively then, damage estimations produced by TFF application methods will produce different results depending on the TFF applied. This begets the following: how different are these results? Hence, how does one choose an appropriate TFF to apply? In light of the proliferation of TFF studies, particularly under the lens of climate change aggravated risk, we believe that exploring the application of these models will be beneficial to the disaster management community at large; beyond the capacity to describe a disaster in the context of other disasters. Considering the research gaps described in the previous section, we:

1. test TFF application methods proposed in the literature and evaluate their performance using common classification metrics against a known dataset;
2. investigate different experimental scenarios applying TFF methods to produce building damage estimates;
3. test a machine learning model as a building damage estimator, training it to recognize damage patterns, and learning from demand parameters inspired by tsunami fragility functions. All our tests are performed on a subset of the well-studied 2011 Great East Japan Earthquake tsunami damage dataset (Fig. 1)^34,35^ to promote clarity in our experimental results.

## Results

### Setup

We conduct experiments on a subset of the 2011 Tohoku Earthquake and Tsunami MLIT dataset^34^, specifically the Ishinomaki dataset (dataset 305 in the convention adopted by MLIT), but we prepare the Sendai plains dataset (320) and the Rikuzentakata dataset (212) the same way; the latter two datasets are used to train the machine learning model (Fig.  1). Three TFFs from the literature are tested: Koshimura et al.^1^, Suppasri et al.^21^, and Belliazzi et al.^25^. Because Koshimura et al.^1^ only maps two damage states, it is excluded from the multi-class experiments. We resample the damage states from the original MLIT 7 classes into binary and 3-class datasets using the following mappings:

1. : $$DS_{MLIT} = \{6,5,4,3,2,1,0\} \mapsto DS_{*} = \{1,1,0,0,0,0,0\}$$ and
2. : $$DS_{MLIT} = \{6,5,4,3,2,1,0\} \mapsto DS_{*} = \{1,1,2,2,2,3,3\}$$.

Our rationale is given in “Data and experimental setup” subsection.

**Figure 2:**
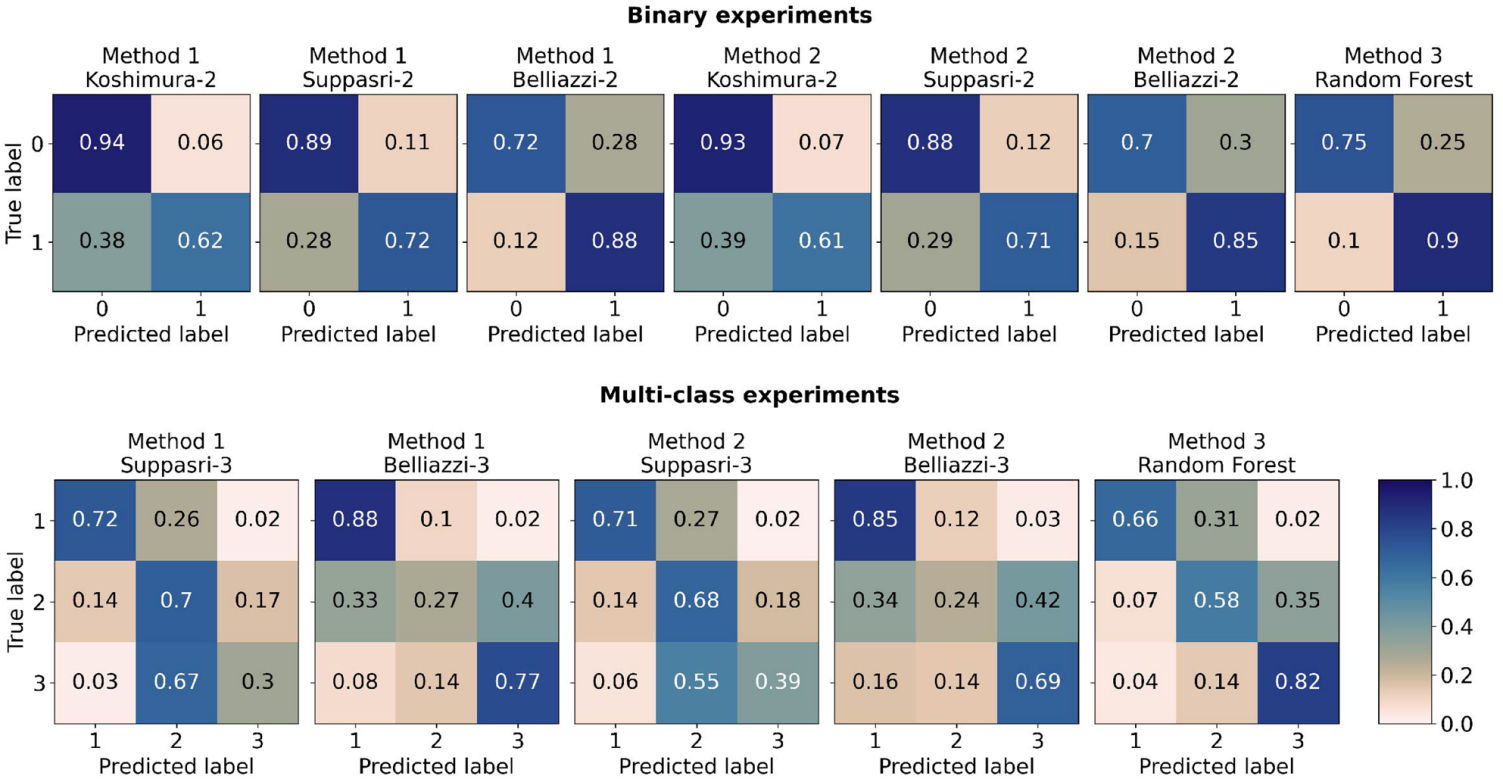
Top, left to right: confusion matrices for binary experiments Method 1^45^ for fragility functions developed by Koshimura et al.^1^, Suppasri et al.^21^, and Belliazzi et al.^25^; Method 2^46^ for fragility functions developed by Koshimura et al.^1^, Suppasri et al.^21^, and Belliazzi et al.^25^; Method 3, binary damage estimation using our proposed method. Bottom, left to right: confusion matrices for multi-class experiments Method 1^45^ for fragility functions developed by Suppasri et al.^21^ and Belliazzi et al.^25^; Method 2^46^ for fragility functions developed by Suppasri et al.^21^ and Belliazzi et al.^25^; Method 3, multi-class damage estimation using our proposed method. All plots were generated using Matplotlib 3.7.2 (https://github.com/matplotlib/matplotlib).

Results are benchmarked by reporting the $\hbox {F}_1$-score, precision, and row-normalized the confusion matrices (Fig.  2). We test two TFF estimation methods from the literature and our proposed machine learning method:

1. : described by Adriano et al.^45^,
2. : described by Moya et al.^46^, and
3. : machine learning algorithm implementing a simple random forest classifier.

The details of each method are reported in sections: Methods 1, Methods 2, and Methods 3 and summarized in Fig. 5.

### Binary experiments

Herein are reported the results from binary experiments; for uniformity, each experiment is referenced in terms of the method (Method 1, Method 2, or Method 3) and the fragility function (when relevant) according to the following naming convention: Koshimura-2 (Banda Aceh)^1^, Suppasri-2 (Tohoku)^21^, and Belliazzi-2 (Analytic)^25^. Method 1 (Table 1; Fig. 2) outperforms Method 2 (Table 2; Fig. 2) in all cases. In terms of TFF Suppasri-2 shows the best performance (average $\hbox {F}_1$-score of 0.809 adopting Method 1, Table 7), this is not surprising considering that the TFF designed by Suppasri et al.^21^ models the MLIT dataset, hence it contains the test data. We refer to such a setting as the “in-domain” (ID) test since the training distribution contains the test distribution^47^; we refer to the TFFs proposed by Koshimura et al.^1^ and Belliazzi et al.^25^ as the “out-of-domain” (OOD) models; inherently, assume that the distributions of all three models are *similar*^47^. It is worth noting that even though Suppasri performs overall better, the average $\hbox {F}_1$-scores for all binary cases are within $\pm 0.05$ (Table 7) of each other. On average, Koshimura-2 performs better than Belliazzi-2 on the test set; interestingly, in terms of individual class scores, Koshimura-2 matched Suppasri-2 when predicting DS0, while both are worse than Belliazzi-2 when it comes to DS1, Belliazzi-2 has the least inter-class variance out of all models (Table 7). Method 3 performs slightly worse on average, due to underestimating DS0 overall but outperforms other methods in DS1. Moreover, Method 3 has the smallest inter-class deviation (Table 7) and displays minimal randomness across runs (average of $0.5\%$, Table 3).

**Table 1:** Metrics of performance evaluation for each binary TFF using application Method 1^45^.

Damage state	Koshimura-2 (Banda Aceh)		Suppasri-2 (Tohoku)		Belliazzi-2 (Analytic)	
	Precision	$\hbox {F}_1$-score	Precision	$\hbox {F}_1$-score	Precision	$\hbox {F}_1$-score
DS 0	0.777	0.850	0.817	0.850	0.892	0.799
DS 1	0.879	0.730	0.820	0.767	0.696	0.776

**Table 2:** Metrics of performance evaluation for each binary TFF using application Method 2^46^.

Damage state	Koshimura-2 (Banda Aceh)		Suppasri-2 (Tohoku)		Belliazzi-2 (Analytic)	
	Precision	$\hbox {F}_1$-score	Precision	$\hbox {F}_1$-score	Precision	$\hbox {F}_1$-score
DS 0	0.770	0.842	0.806	0.820	0.862	0.772
DS 1	0.860	0.715	0.803	0.751	0.669	0.746

**Table 3:** Metrics of performance evaluation for binary random forest classifier and stability of metric over 30 seeds for binary random forest classifier.

Class	Precision	$\hbox {F}_1$-score	$\mu _{F_1} \pm \sigma _{F_1}$
DS 0	0.912	0.790	$0.796 \pm 0.006$
DS 1	0.687	0.782	$0.786 \pm 0.004$

$\mu _{F_1}$ and $\sigma _{F_1}$ are respectively the mean and standard deviation of the $\hbox {F}_1$-score over 30 seeded runs.

**Figure 3:**
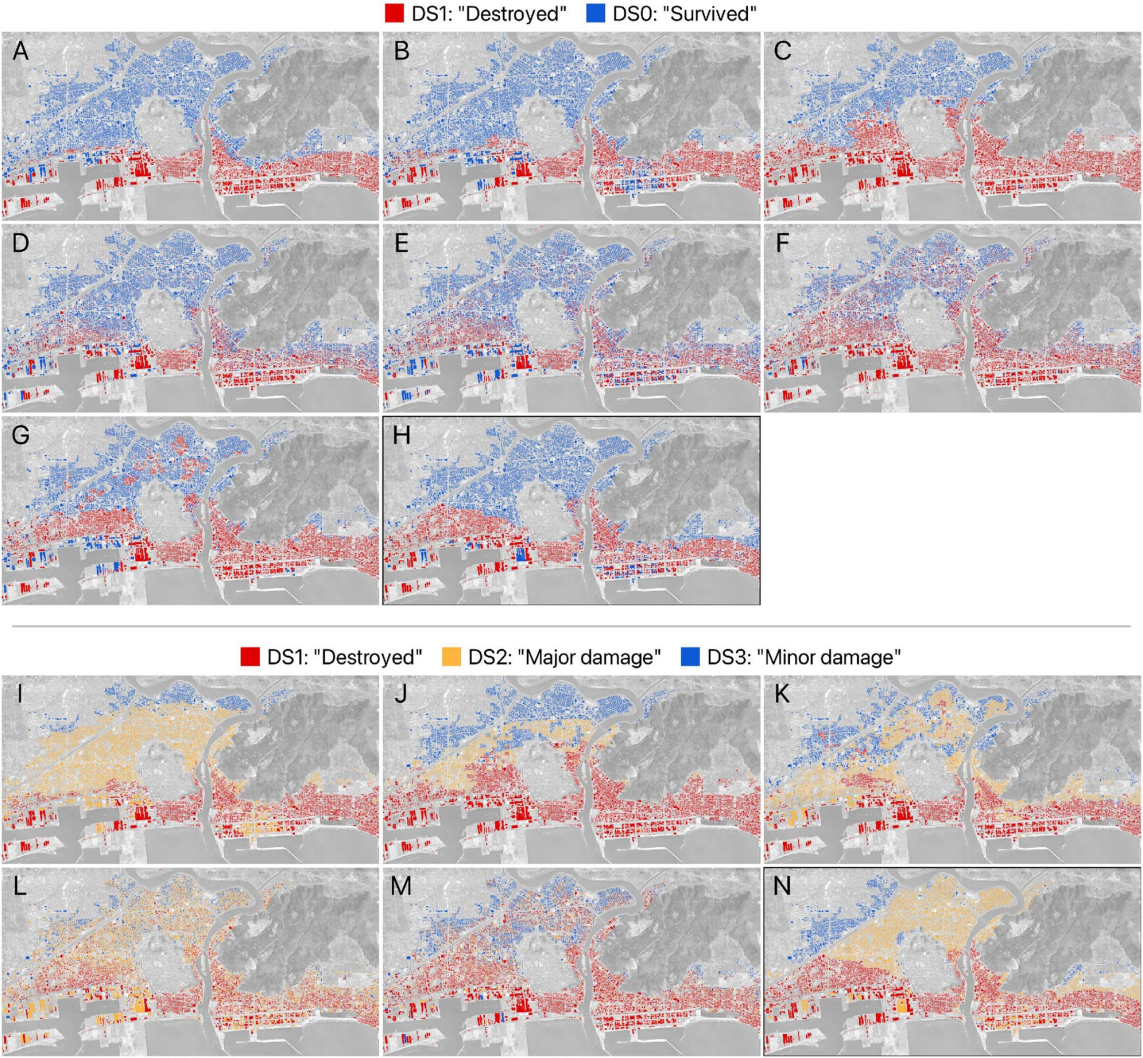
Results plotted spatially. Binary results above line: (**A**) Method 1^45^, Koshimura-2^1^; (**B**) Method 1, Suppasri-2^21^; (**C**) Method 1, Belliazzi-2^25^; (**D**) Method 2^46^, Koshimura-2; (**E**) Method 2, Suppasri-2; (**F**) Method 2, Belliazzi-2; (**G**) Random forest classifier; (**H**) Ground truth (binary). Multi class results below line: (**I**) Method 1^45^, Suppasri-3^21^; (**J**) Method 1, Belliazzi-3^25^; (**K**) Random forest classifier; (**L**) Method 2^46^, Suppasri-3; (**M**) Method 2, Belliazzi-3; (**N**) Ground truth (multi-class) Original maps generated using QGIS 3.28.2-Firenze (https://qgis.org/en/site).

Spatially (Fig.  3), a few key differences between the methods become evident: Method 1 (Fig.  3; frames A–C) appears very strongly clustered, ostensibly due to the ordering imposed on it, which also removes any randomness; Comparing the best performer, Suppasri-2 (Fig.  3, Frame B) to the ground truth (Fig.  3, Frame H) the inland threshold between damage states is understated on the westward and overstated eastward of the estuary additionally much of the nuanced damage along the foreshore is not represented in the model. The most evident feature of Method 2 (Fig. 3; frames D–F) is the spatial scattering due to the random component of this method which is not immediately evident from the metrics alone. Unlike the previous method, the boundary between damage states is much less defined, though it approximates the boundary drawn by the ground truth more than the previous method. As a result of the inherent randomness, the damage along the foreshore is much more nuanced, and closer to the ground truth on the east side. Moreover, due to the random scattering and contrary to reality, damage is much more sparse and less clustered. Method 3 (Fig.  3; frame G) like Method 1 draws a much cleaner boundary between damage states. Interestingly, it mistakenly identifies several clusters of damaged buildings significantly inland of the coast. However, it is much more faithful to the ground truth along the estuary and harbor preserving some of the nuanced damage in this area. Performance is worse along, and inland of, the east coast where much of the nuance along the foreshore is lost, similar to Method 1.

### Multi-class experiments

Herein are reported the results for the multi-class experiments; as above, each experiment is referenced in terms of the method (Method 1, Method 2, or Method 3) and the fragility function (when relevant) according to the following naming convention: Suppasri-3 (Tohoku)^21^, and Belliazzi-3 (Analytic)^25^. As mentioned, Koshimura et al.^1^ is not applicable to multiple damage states and is therefore excluded.

**Table 4:** Metrics of performance evaluation for each multi-class TFF using application Method 1^45^.

Damage state	Suppasri-3 (Tohoku)		Belliezzi-3 (Analytic)	
	Precision	$\hbox {F}_1$-score	Precision	$\hbox {F}_1$-score
DS 1	0.820	0.767	0.696	0.776
DS 2	0.622	0.657	0.673	0.385
DS 3	0.310	0.304	0.334	0.467

**Table 5:** Metrics of performance evaluation for each multi-class TFF using application Method 2^46^.

Damage state	Suppasri-3 (Tohoku)		Belliezzi-3 (Analytic)	
	Precision	$\hbox {F}_1$-score	Precision	$\hbox {F}_1$-score
DS 1	0.805	0.756	0.665	0.743
DS 2	0.628	0.651	0.607	0.342
DS 3	0.352	0.370	0.299	0.418

**Table 6:** Metrics of performance evaluation for multi-class random forest classifier and stability of metric over 30 seeds for multi-class random forest classifier.

Class	Precision	$\hbox {F}_1$-score	$\mu _{F_1} \pm \sigma _{F_1}$
DS 1	0.885	0.758	$0.750 \pm 0.005$
DS 2	0.638	0.608	$0.612 \pm 0.007$
DS 3	0.378	0.518	$0.521 \pm 0.006$

$\mu _{F_1}$ and $\sigma _{F_1}$ are respectively the mean and standard deviation of the $\hbox {F}_1$-score over 30 seeded runs.

In the multi class experiment, overall performance of fragility curves is markedly lower across all metrics (Table 7). This time Method 3 markedly outperforms the other two methods, mainly due to TFF methods’ poor significantly worse performance across DS2 and DS3. Class-wise, Method 1 and Method 2 still perform marginally better than Method 3; this is not surprising since DS 1 remains unchanged between the binary and multi-class experiments. Across both Method 1 (Table 4) and Method 2 (Table 5 Suppasri-3 performs significantly lower than Belliazzi-3 in DS3, conversely Suppasri-3 outperforms Belliazzi-3 across DS2 almost doubling the $\hbox {F}_1$-score in most cases. DS1 is slightly more uncertain with the best performer being Belliazzi-3 modelled by Method 1. Method 3 continues to have the least inter-class deviation (Table 7) while still offering a relatively stable estimator as shown in Table 6. Looking at the confusion matrices (Fig.  2), and noting that the recall (i.e., the fraction of true positives and positive values) is given by the main diagonal, it appears that, in spite of the metric, DS2 is the more problematic across all TFF methods for the multi class case, though the error mode differs between Suppasri-3 (Tohoku) and Belliazzi-3 (Analytic). More specifically, in Suppasri-3 (Tohoku) the largest error rate is for type-I errors, i.e., a DS2 false positive error, while in applications of Belliazzi-3 (Analytic) the largest error rate is for type-II errors, i.e., a DS2 false negative error. In Method 3 it is not quite as clear, though the recall (true positive rate) across all classes remains above the false positive rate and false negative rate. It seems apparent that for TFF methods (Method 1 and Method 2) the middle class (DS2) remains particularly ambiguous, in terms of the inundation depth and distance from the coast alone. It is possible that increasing the dimensionality of the problem may allow for a linear classifier to separate the classes. Spatially, many of the trends displayed by the binary experiments are evident: Method 1 (Fig. 3, frames I,J) continues to lose a lot of nuance towards the coast while wrongly estimating the damage boundaries, particularly DC3, which seems to be limited to the North-West in the ground truth (Fig.  3, frame N), is poorly estimated by either TFF. Method 2 (Fig.  3, frames J,M) maintain a lot of randomness, which is especially marked in Belliazzi-3 where DS2 is virtually inseparable from the other states, Suppasri-3 still shows a lot of uncertainty across DS2, but allows for the distinction of likely, albeit vague, boundaries. Method 3 (Fig.  3, frame K) still loses a lot of nuance along the foreshore, estimating virtually all samples as DS1, but approximates the DS1–DS2 boundary more closely than other experiments on the East side, while failing to do so on the far West side; a lot of the characteristic isolated clusters are present and scattered across the main DS2 body from both DS1 and DS3, which is mirrored in the false negative rate in the confusion matrices (Fig.  2). Method 3 overestimates DS3 by a large margin, however it better represents the north-south extent of the class, while encroaching significantly into DS2.

## Discussion

**Figure 4:**
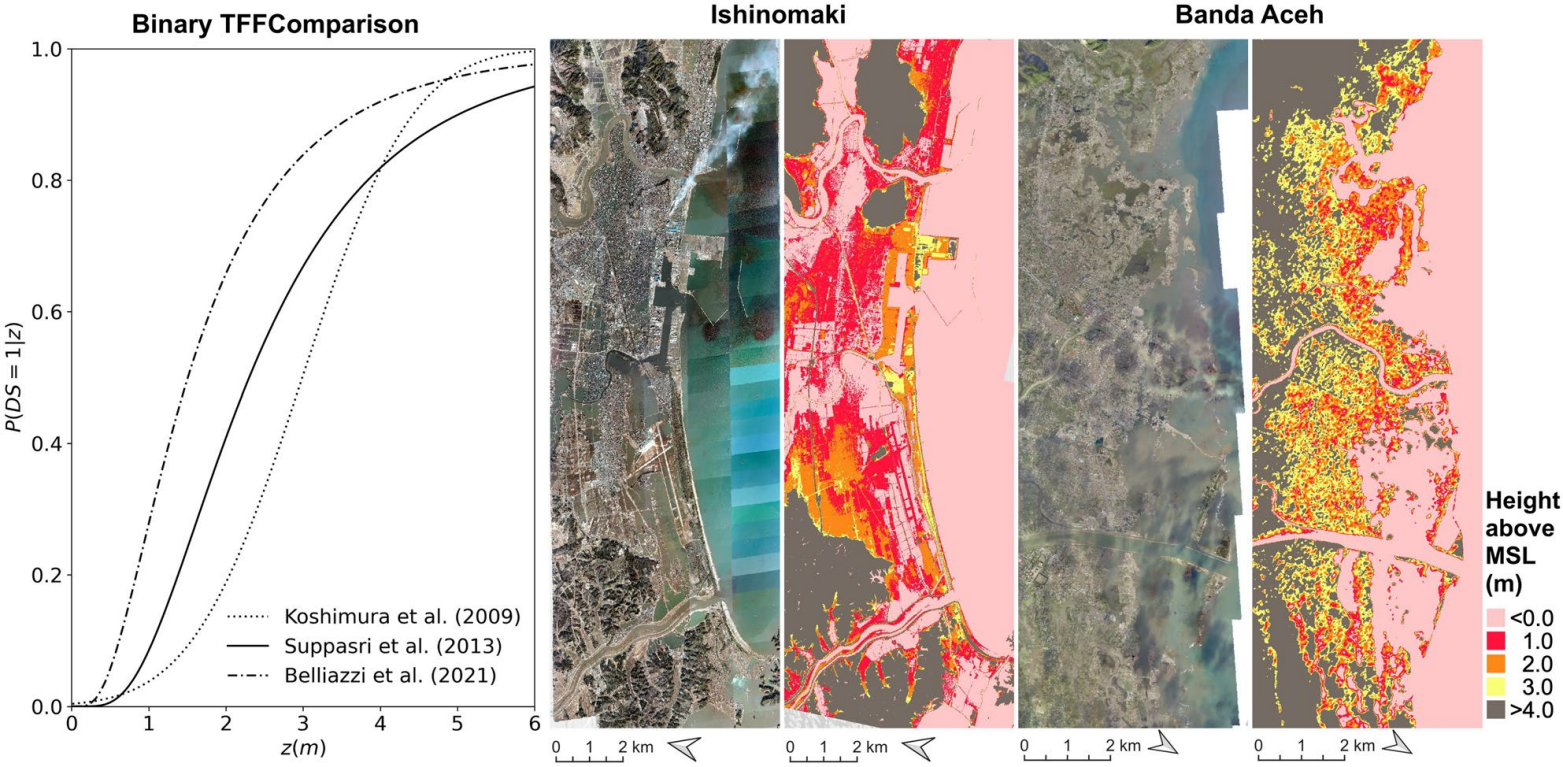
From left to right: comparison between binary TFFs employed in the present experiments expressing the probability of buildings being destroyed $P(DS1 \mid z)$ when $z \in [0, 10]$ generated using Matplotlib 3.7.2 (https://github.com/matplotlib/matplotlib); aerial imagery after the 2011 Great East Japan Tsunami and DEM of Ishinomaki (Created by processing Geospatial Information Authority of Japan tiles—elevation tiles^49^); aerial imagery after the 2004 Indian Ocean Tsunami and DEM of Banda Aceh^50^ (elevation models clipped at 1, 2, and 3 m above MSL). Original maps generated using QGIS 3.28.2-Firenze (https://qgis.org/en/site).

We set out to test the application of tsunami fragility functions for tsunami damage estimation, their transferability, and test their limitations. Additionally, we propose a supervised classifier alternative using canonical TFF demand parameters in the feature matrix (complemented by several others) generated from the pre-disaster landscape. Herein, we discuss the implications of our findings. Binary, out-of-domain experiments generally performed within a 6.75% (Table 7) margin of each other (centered around 78.02%) on the metric. This suggests that some level of generalization is achieved by these methods. It is unlikely that the present results are representative of all TFF estimations and further testing is encouraged. We refer the reader to Mas et al.^8^ wherein 6 binary fragility functions are discussed; the study compares the various probabilities at certain threshold values. Our results exemplify the variability between a foreign TFF when compared to a “True” frame of reference, in this case the difference between Belliazzi’s^25^ or Koshimura’s^1^ (OOD) curves relative to the “more appropriate” TFF by Suppasri et al.^21^ (ID). Spatially, this variability presents itself as a “shift” between class interfaces; we postulate that testing further TFFs will generate damage maps where the damage interface is again shifted. Inherently, for any given domain affected by a tsunami, the “best” TFF will be the one that approximates the real damage interface the most. By contradiction, the ability to conduct such a comparison presupposes the availability of a local TFF. In the absence of this, the pertinence of a TFF will depend on the similarity between the training domain and the target domain^47^, hence meaningful applications of these methods to future or potential domains will hinge on appropriate assessment of TFF suitability. It is interesting therefore, that Koshimura et al.^1^ performs surprisingly well in terms of the $\hbox {F}_1$-score. Koshimura et al.^1^ report that the topography of the center of the city is low-lying with elevations, lower than 3 m above mean sea level (MSL). Further, most buildings in the tsunami-affected area were low-rise wooden house, timber construction, and non-engineered reinforced concrete (RC). The tsunami was found to have penetrated 3–4 km inland throughout the city, with inundation up to 7–9 m along the western coast. Comparatively, Suppasri et al.^48^ report that tsunami heights along the Ishinomaki coasts were more than 10 m, while inundation depths in populated areas were more than 5 m. In Japan, wooden houses are preferred to reduce the earthquake impact due to the lighter frame. The authors identify inundation depth above 2 m MSL to be highly correlated to severe damage to these types of structure. From Fig. 4 we can observe that 50% of the Destroyed buildings occur approximately at $z > 2$ m in the TFF proposed by Suppasri et al.^21^ and approximately at $z > 3$ m in the TFF proposed by Koshimura et al.^1^. The digital elevation models for each domain reveal that major portions of both settlements lie below 4 m (above the local vertical datum). From the satellite imagery we can additionally observe that a vast portion of structures lay within 3–4 km from the coast and almost entirely within the flood extent. The Fragility functions suggest that buildings in Banda Aceh may be slightly less susceptible to inundation, illustrated by the smaller initial gradient in Koshimura et al.^1^’s TFF. This is corroborated in Fig. 3 (Frames A,B), in which estimations using Koshimura-2^1^ produce an interface closer to the coast, than what is produced by Suppasri-2^21^. Notwithstanding, significant similarities in geomorphology, building material distribution, and building arrangement may explain the performance of Koshimura-2^1^ on the metric (Tables 1, 2).

**Table 7:** Average $\hbox {F}_1$-scores for binary and multi class experiments.

Experiment	Average $\hbox {F}_1$-score
Koshimura-2 (Banda Aceh), Method 1	$0.790 \pm 0.060$
Koshimura-2 (Banda Aceh), Method 2	$0.779 \pm 0.063$
Suppasri-2 (Tohoku), Method 1	$0.809 \pm 0.041$
Suppasri-2 (Tohoku), Method 2	$0.795 \pm 0.045$
Belliazzi-2 (Analytic), Method 1	$0.788 \pm 0.011$
Belliazzi-2 (Analytic), Method 2	$0.759 \pm 0.013$
Random Forest Classifier (binary)	$0.786 \pm 0.003$
Suppasri-3 (Tohoku), Method 1	$0.576 \pm 0.197$
Suppasri-3 (Tohoku), Method 2	$0.593 \pm 0.163$
Belliazzi-3 (Analytic), Method 1	$0.543 \pm 0.168$
Belliazzi-3 (Analytic), Method 2	$0.501 \pm 0.174$
Random Forest Classifier (multi-class)	$0.628 \pm 0.099$

Method 3 on the other hand allows for additional dimensionality to be added to the problem; while it does not outperform the specific TFFs tested in this study, may allow better estimates if the training set is extended to different, well documented, domains. Additionally, it is important to consider potential use cases: for example, in disaster relief cases, the randomness outputted by Method 2 would be detrimental as it does not restrict the estimated damage extent. In the context of a routing problem for example, in which an agent must check all DS1 buildings to provide supplies^51^, the agent would have to cover significantly more ground when applying estimates using Method 2 compared to the other 2 methods. The significant loss in performance in the multi-class experiments suggests that TFF are generally unreliable multi-class damage estimation; it is possible that further research, such as evaluating TFF derived from generalized linear models or developing entire new methods to apply these models could yield more reliable estimates. Especially ones that allow for more parameters to be considered. By the same token, further testing of machine learning methods with a larger set of learnable parameters may enable estimators that reliably generalize.

## Conclusion

In this study we perform tsunami building damage estimation in Ishinomaki City, Japan, after the 2011 Great East Japan Earthquake using physical parameters—our tests are evaluated against the post disaster survey ground truth^34^. We compare three methodologies: two TFF application methods from the literature^45,46^ and a machine learning model trained on out-of-domain physical parameters; Hence, we perform binary and multi-class experiments. In the case of TFF applications, we select three tsunami fragility functions (two in the multi class tests) to check the variability between the application of in-domain TFFs versus out-of-domain TFFs. We verify that TFF application methods are able to produce building damage estimation with variable success. The performance of TFF estimates is contingent on both the method and the suitability of the TFF to the domain (due to latent variables obfuscated by the demand parameters, see previous section): of our attempted methods, Method 1 is consistently superior to Method 2 on both the metric and spatially for all cases. The best performing TFF is unsurprisingly the in-domain case^21^, though out-of-domain TFFs in the interval $78.02\pm 6.65\%$ for the binary case. In addition to testing TFF application methods, we propose an novel re-interpretation of damage estimation based on physical parameters. We draw inspiration from TFF demand parameters and propose a machine learning framework: Method 3 produces comparable results to TFF methods in the binary tests but has improved performance in the multi-class case suggesting greater flexibility. With this study, we contribute novel methods to generate tsunami damage estimations at the building scale to inform disaster risk managers of potential risk areas during and after tsunamis. Our method is applicable to simulated inundation scenarios, such as those envisioned by probabilistic tsunami hazard assessments, hence could provide greater detail in disaster planning and preparedness tasks. In future we plan to investigate alternative methods to apply TFFs to damage estimation problems at the building scale. Moreover, it is our hope that the proposed methodology will inspire further studies to investigate machine learning methods that learn and generalize better to different domains.

## Methods

**Table 8:** Features used to train the random forest classifier.

Variable	Description
*DS*	Rank value representing relative degree of damage (Label)
$C_{bld}$	Building material classification
$h_{datum}$	Vertical elevation of building above datum
$z_{ground}$	Depth of inundation w.r.t. ground
$\rho _{bld}$	Kernel density estimation of buildings with uniform radius
$d_{coast}$	Euclidean distance from coast including coastal protection structures
$d_{water}$	Euclidean distance from closest body of water

Feature extraction and derivation is elaborated in the “Physical parameters” section.

### Data and experimental setup

We conduct experiments on a subset of the 2011 Tohoku Earthquake and Tsunami MLIT dataset^34^, specifically the Ishinomaki dataset (codifies as dataset 305) but we prepare the Sendai plains dataset (320) and the Rikuzentakata dataset (212) the same way; the latter two datasets are used to train the machine learning model.

#### Physical parameters

Out of the box, the dataset includes several of the features needed: building material $C_{bld}$, inundation above ground level $z_{ground}$, topographic elevation w.r.t. the datum $h_{datum}$, and the damage state (label/dependent variable) *DS*. The remaining features were computed spatially: the building density $\rho _{bld}$ is taken as the two dimensional kernel density estimation between building centroids, for the purposes of this experiment we used a constant radius (500 m) KDE calculated using QGIS. The distance from the coast $d_{coast}$ and the distance from sheltered waters $d_{water}$ are taken as the euclidean distance between the building centroid and the respective coastline. $d_{coast}$ is drawn to include coastal protection measures such as seawalls, groynes, breakwaters, and does not track inland via rivers or ports. $d_{water}$ instead tracks the absolute land–water interface including sheltered waters, rivers, and ports. The distinction was drawn to investigate the relative impact of coastal protection structures. A summary of the physical parameters is provided in Table 8. It is important to note that damage sustained by any given building, as a result of a tsunami generated by a megathrust earthquake, is generally subjected to loads generated by the earthquake itself, in addition to those imposed by the tsunami. Seismically derived loads can include, but are not limited to, strong ground motion, soil liquefaction, collapse or surrounding environment (built and natural), etc; the seismic activity that is principally the cause of earthquake loads (and generation of the tsunami itself) is dependent on geological processes in the lithosphere, such as slope stability, tectonic subduction, asperity along the fault, etc. It is acknowledged that building damage is directly influenced by these factors and processes and tsunami intensity measures (engineering demand parameters, and several physical parameters that are used in the present study) are generally consequent upon these factors. Geological and seismic characteristics are used to set the initial condition of the hydrodynamic modelling that generates the intensity measures—hence, they are intrinsic to the inundation depth and further demand parameters. Direct effects of seismic and geological characteristics are ultimately not included in the machine learning training; there are a few reasons for this decision: (1) initial tests using seismic characteristics yielded inferior results to the ones reported above; this is perhaps due to the spatial coarseness that ultimately results in sparse data. (2) They are not directly included in the original modelling of the empirical fragility functions tested in this study.

#### Damage states

The damage state *DS* is graded in 7 ranks, from least to most damage: “no damage”: DS0, “partial damage”: DS1, “50% damage”: DS2, “50–70% damage”: DS3, “1st level destroyed and flooding above”: DS4, “completely destroyed”: DS5, “washed away”: DS6. While these classifications are meaningful in a structural engineering context, they might not reflect entirely on the *EDP*’s. Moreover, only TFFs based on the GEJE damage are built on 7 damage states; hence, it is necessary to simplify the classification so that it is comparable. The following mappings are used to translate the original MLIT classification into the target classes for each experimental setup (see next section):

1. : $$DS_{MLIT} = \{6,5,4,3,2,1,0\} \mapsto DS_{*} = \{1,1,0,0,0,0,0\}$$ and
2. : $$DS_{MLIT} = \{6,5,4,3,2,1,0\} \mapsto DS_{*} = \{1,1,2,2,2,3,3\}$$ .

Succinctly, DS6 and DS5 are combined in Mapping 1 as Koshimura et al.^1^ and Belliazzi et al.^25^ do not distinguish between completely destroyed and washed away. We extend this convention to Mapping 2 for consistency. The other groupings in Mapping 2 were decided by testing all other possible 3-class groupings that complied with the previous condition, and selecting the mapping that overall performed best on the metric.

**Figure 5:**
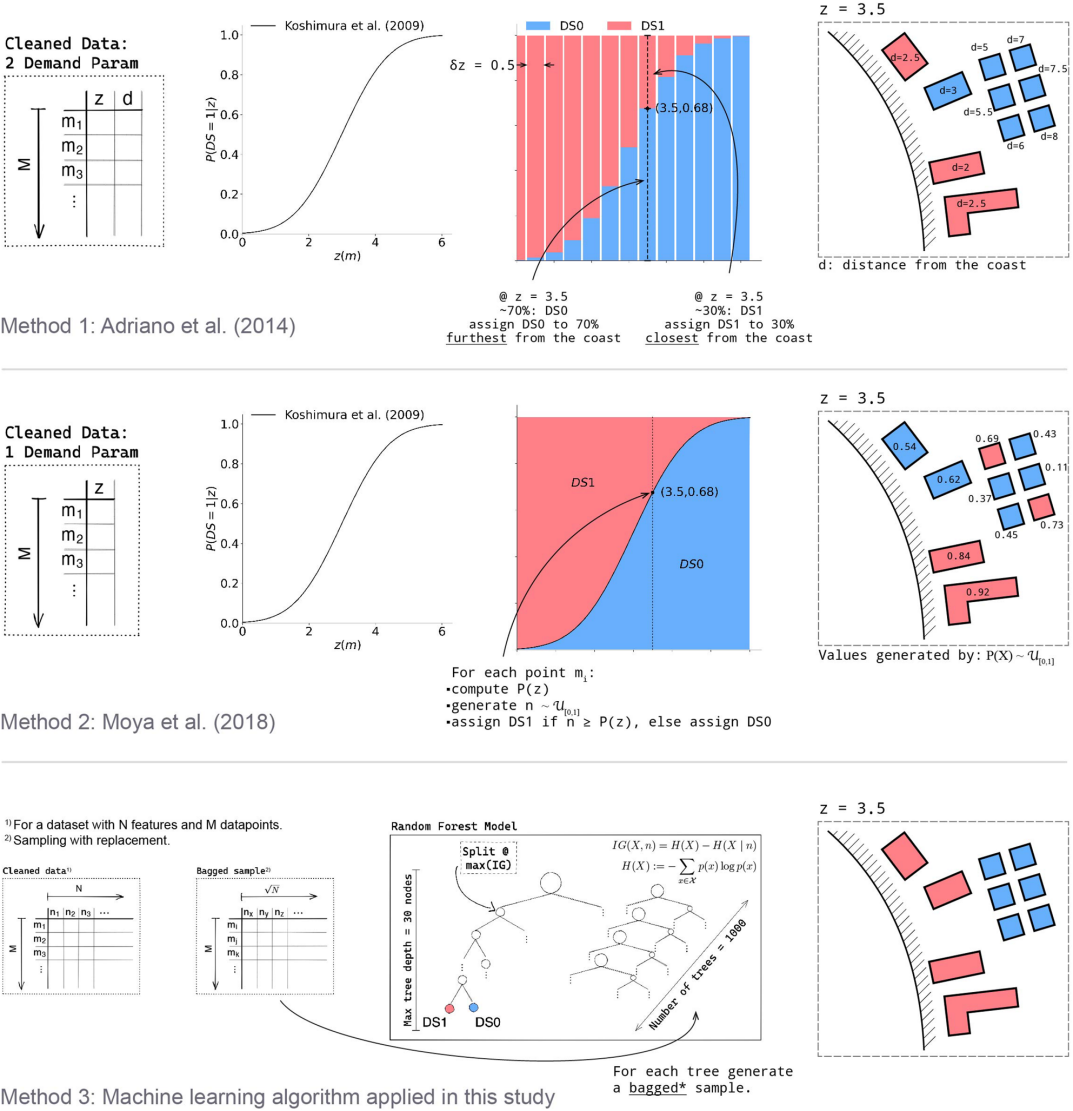
Top: visualization of framework proposed by Adriano et al.^45^. Middle: visualization of framework proposed by Moya et al.^46^. Bottom: visualization of framework proposed in the present study. Original figure generated using Adobe Illustrator 27.7, Excalidraw 0.15.0 (https://github.com/excalidraw/excalidraw), and Matplotlib 3.7.2 (https://github.com/matplotlib/matplotlib.

#### Experimental setup

The experimental setup consists of a set of binary experiments and a set of multi-class experiments were two TFF application methods are tested for each set. Because Koshimura et al.^1^ only maps two damage states, it is excluded from the multi-class experiments. Three TFFs from the literature are tested: Koshimura et al.^1^, Suppasri et al.^21^, and Belliazzi et al.^25^. Moreover, we propose and test an alternative to TFF applications using a simple machine learning classifier (random forest) and perform the same estimation tasks (one binary, one multi class) by expanding the demand parameter matrix to include additional dimensionality. We benchmark our results by reporting the $\hbox {F}_1$-score, Precision, and row normalized the confusion matrices (Fig.  2). We test two TFF estimation methods from the literature and our proposed machine learning method:

1. : described by Adriano et al.^45^,
2. : described by Moya et al.^46^, and
3. : machine learning algorithm implementing a simple random forest classifier.

### Tsunami fragility function applications for damage mapping

Fragility functions express the probability that a building under a specific load, parameterized as an intensity measure or demand parameter, will achieve a specific damage state. OLS tsunami fragility functions are generally fit to the cumulative distribution function (CDF) of a statistical model belonging to the exponential family: most often the normal (Eq. 1) or lognormal (Eq. 2) distributions. These can be expressed symbolically as:

1 $$\begin{aligned} F_{X,i,DS} (x)= & {} \Phi \left[ \frac{x - \mu _{i,DS}}{\sigma _{i,DS}} \right] = \frac{1}{2} \left[ 1 + {{\,\textrm{erf}\,}}\left( \frac{x - \mu _{i,DS}}{\sigma _{i,DS} \sqrt{2}}\right) \right] \end{aligned}$$

2 $$\begin{aligned} F_{X,i,DS} (x)= & {} \Phi \left[ \frac{\ln x - \mu _{i,DS}}{\sigma _{i,DS}} \right] = \frac{1}{2} {{\,\textrm{erfc}\,}}\left[ - \frac{\ln x - \mu _{i,DS}}{\sigma _{i,DS} \sqrt{2}}\right] \end{aligned}$$

Hence we define:

3 $$\begin{aligned} P_{i,DS}(DS=ds \mid X=x) = F_{X,i,DS}(x\mid \mu _{i,DS},\sigma _{i,DS}) \end{aligned}$$

Where $F_X(x)_{i,DS}$ is the cumulative distribution function for $\text {ln}(X) \sim N(\mu _{i,DS}, \sigma ^2_{i,DS})$, $\Phi$ is the CDF of the standard normal distribution $N(0, 1)$ and ${{\,\textrm{erfc}\,}}$ is the complementary Gauss error function ${{\,\textrm{erfc}\,}}{z} = 1 - {{\,\textrm{erf}\,}}{z} = 1 - \frac{2}{\sqrt{\pi }}\int _0^z e^{-t^2}\,dt$. While $\mu _{i,DS}$ and $\sigma ^2_{i,DS}$ are the mean and variance of the distribution for building class *i* and damage state *DS*. The random variable *X* represents the demand parameter, in this case we adopt the inundation depth *z*.

### Method 1

The method proposed by Adriano et al.^45^ (Fig. 5, Top) requires the data to be sorted in ascending order by a parameter different from the main demand parameter *z*, in this case we choose the distance from the coast $d_{coast}$. An interval of inundation depths is chosen to subdivide the data (in our case 0.5 m). The data is split into subgroups such that each subgroup contains all data that is within the interval, i.e., all data points that have inundation depths $0 \, m \le z < 1 \, m$ are in one subgroup, points that have inundation depths $1 \, m \le z < 2 \, m$ are in another, et cetera. For each subgroup, the mean depth $\mu _z$ is calculated. Given the set of target damage states, such as $DS: \{0,1\}$ and a set of TFF, $F_{X,DS}(x)$, that maps $z \mapsto P(DS = ds)$ we generate $P(DS = ds \mid x = \mu _z)$ for $ds \in DS$ to obtain the proportion of buildings in the interval that belong to each damage state^1^. The damage state is assigned based on the ordering of the secondary parameter (as before, this is defined as the distance from the coast $d_{coast}$) by making an assumption about the nature of the ordering relative to the damage state: explicitly, it is assumed that buildings closer to the coast $d_{coast}$ are more likely to be damaged by a tsunami.

### Method 2

The method proposed by Moya et al.^46^ (Fig. 5, middle) establishes that the probability of a building subject to a demand parameter to achieve damage state $P(DS\ge ds \mid X=x)$ is given by Eq. (4):

4 $$\begin{aligned} P (DS \ge ds \mid X = x) = {\left\{ \begin{array}{ll} 1 - F_{X,ds}(x) &{}: ds = 0 \\ F_{X,ds}(x) - F_{X,ds+1}(x) &{}: 1 \le ds < i\\ F_{X,i}(x) &{}: ds = i \end{array}\right. } \end{aligned}$$

Consequently, for each building subject to *z* we calculate the vector of probabilities ${\varvec{P}} = [P_{0}, P_{1}, \ldots , P_{i}]$, noting that $|{\varvec{P}}|= 1$. We generate a uniformly distributed random number $Y \sim {\mathscr {U}}_{[0,1]}$ and check $Y \le {\varvec{P}}$ element-wise. Each point is assigned the least possible damage state out of the all damage states that satisfy the inequality.

### Method 3

We propose a feature-extracted simple machine learning classifier (Fig.  5, bottom) alternative to the TFF estimation methods to: (1) create a baseline, (2) verify whether damage estimation can be approached using engineering quantities, and (3) benchmark the performance of TFF estimation methods. As explained briefly in “Results”, the feature matrix for the machine model is populated with well studies quantities in the TFF literature and additional quantities synthesized from remote sensing and survey data. The motivation to develop two different horizontal distances stems from wanting to characterize tsunami surge travelling deeper inland via water channels as highlighted in tsunami engineering literature^52^. Building density $\rho _{bld}$ is calculated using kernel density estimation with an arbitrary radius of 500 m for each building. Features are individually centered and scaled by the interquartile range to avoid outlier bias. The labels are reclassified into 2 (binary, Mapping 1) and 3 (multi-class, Mapping 2) classes in order to obtain estimates comparable to those produced by the TFF methods. In this instance we use entropy to measure the purity of our nodes, while other hyperparameters are reported in Table 9. The model is trained on the Sendai city and Rikuzentakata subsets of the MLIT data and tested on the unseen Ishinomaki data set, hence we only test the out-of-domain case for both binary and multi class scenarios.

**Table 9:** Random forest hyperparameters for a feature matrix of size $m \times n$.

Hyperparameter	Value
Number of estimators (trees)	1000
Maximum tree depth	30
Minimum samples in leaf	1
Minimum number of samples to split impure nodes	2
Maximum number of features used in each split	$\sqrt{n}$
Maximum number of data points per bootstrap	*m*

## Data Availability

Data informing the present findings is available on reasonable request. Further, building damage data for the 2011 Great East Japan Earthquake is also publicly avaiable at http://fukkou.csis.u-tokyo.ac.jp/dataset/list_all and https://www.mlit.go.jp/toshi/toshi-hukkou-arkaibu.html.
